# Motion capture-based animated characters for the study of speech–gesture integration

**DOI:** 10.3758/s13428-019-01319-w

**Published:** 2019-12-10

**Authors:** Jens Nirme, Magnus Haake, Agneta Gulz, Marianne Gullberg

**Affiliations:** 1 Lund University Cognitive Science, Lund, Sweden; 2 Centre for Languages and Literature, Lund University, Lund, Sweden

**Keywords:** Cross-modal information processing, Gesture, Speech–gesture integration, Motion capture

## Abstract

Digitally animated characters are promising tools in research studying how we integrate information from speech and visual sources such as gestures because they allow specific gesture features to be manipulated in isolation. We present an approach combining motion capture and 3D-animated characters that allows us to manipulate natural individual gesture strokes for experimental purposes, for example to temporally shift and present gestures in ecologically valid sequences. We exemplify how such stimuli can be used in an experiment investigating implicit detection of speech–gesture (a) synchrony, and discuss the general applicability of the workflow for research in this domain.

## Introduction

Gestures are an integral part of natural language use. Gestures, defined as (mostly manual) movements related to the expressive effort and recognized as being communicatively relevant (Kendon, 2004; McNeill, 1992), are prevalent in communication (face-to-face or in groups, e.g., Kendon, 2004; Özyürek, 2002), whether interlocutors are visible or not (Bavelas, Gerwing, Sutton, & Prevost, 2008). Proponents of contemporary gesture theories generally agree on the tight link between speech, language and gestures despite theoretical divides as to the precise nature of the link (De Ruiter, 1998; Hostetter & Alibali, 2008; Kelly, Özyürek, & Maris, 2009; Kita, Alibali & Chu, 2017; Krauss, Chen, & Gottman, 2000; McNeill, 2005). Speech and gestures are seen as forming an integrated whole, where both parts are relevant. The connection is reflected in the close temporal and semantic coordination between gestures and spoken utterances found in language *production* (Kendon, 2004 for an overview) whereby gestures and speech tend to express closely related meaning at the same time. Speech and gestures co-occur and gesturing in the absence of speech is rare in normal conversation. Moreover, speakers gesticulate significantly less during speech disfluencies or pauses than during fluent speech (Graziano & Gullberg, 2018; McNeill, 1985; McNeill, 2005, pp. 34–27). The semantic relationship between speech and gesture has been characterized in terms of ‘lexical affiliates’ (Schegloff, 1984) to denote the word or words whose meaning correspond to that expressed in gestures. However, given that gestures often express ‘imagistic’ information complementing or illustrating the verbally expressed meaning of an utterance as a whole, in its communicative context, the notion of ‘conceptual affiliates’ has been suggested instead (De Ruiter, 2000; see also McNeill, 2005, p. 37).

Turning to *reception*, several studies have demonstrated that addressees understand messages better if they are accompanied by gestures (e.g., Kelly et al., 1999; Rogers, 1978), and conversely, that comprehension is negatively affected if information across modalities is contradictory (e.g., Cassell, McNeill, & McCullough, 1999). Despite the considerable body of research on gestures, some open questions remain concerning speech–gesture integration in language processing. For instance, questions remain as to whether integration is inevitable or flexible, automatic or demanding of mental resources, mirroring patterns in production or better described as a case of general audiovisual integration.

A number of factors are likely to influence integration, some contextual and others related to the kinematic characteristics of the gestures, for example, temporal coordination. The boundary conditions for integration of speech and gestures have yet to be mapped out. One reason for this is methodological in nature, because the implementation of experimental interventions in stimuli poses challenges and often requires trade-offs between control and ecological validity. In order to probe these matters, an experimental platform is needed that enables precise kinematic manipulation of gestures within natural and spontaneous speech and gesture sequences.

In this paper, we present an approach using 3D-animated characters based on Motion Capture (MOCAP, Welch & Foxlin, 2002) data from real speakers, and an experiment to evaluate the method. We will focus specifically on temporal properties of speech–gesture alignment in both the description and evaluation of the method, but also discuss how the method could be applied to other kinematic manipulations of gestures. The question of temporal alignment is relevant for a number of reasons. First, it can potentially reveal some crucial details to how processing in the two channels is integrated and contribute to multimodal language comprehension in real-time. Second, as we will describe below, the available empirical evidence for effects of temporal alignment in language perception is inconclusive. Third, temporal manipulations pose a particular methodological challenge because they are difficult to enact in a natural way and require masking the faces of speakers to avoid confounding reactions to asynchronous lip movements (Massaro, Cohen & Smeele, 1996).

### Speech–gesture coordination in production

It is frequently stated that gestures align temporally with co-expressive spoken elements. Kendon (1972, 1980) found matching parallel hierarchical organizations of concurrent speech and gesture sequences, based on kinematic and prosodic features. Kendon’s organization of gestures into *units* (an ‘excursion’ of one or both hands from and back to a resting position) containing one or more *gesture phrases* consisting of *gesture phases* (preparations, strokes, holds and retractions; Kendon, 2004) has been highly influential. Kendon has also made detailed observations regarding the temporal alignment to the effect that gestural phrases tend to emerge before speech where the same idea is encoded (Kendon, 1980), which led McNeill (1992) to formulate the *Phonological Synchrony Rule* stating that *“[a] gesture precedes or ends at, but does not follow, the phonological peak syllable of speech*” (McNeill, 1992, p. 26).

Even if results differ somewhat depending on operationalizations and units of analysis (Beatty & Aboudan, 1994; Butterworth & Beatty, 1978; Nobe, 2000), findings from studies focused on the alignment of words with strokes, that is, the most effortful and expressive movement phase of a gesture, generally adhere to the phonological synchrony rule, although there is some variation in the degree of ‘anticipation’ of gestures (Chui, 2005; Ferré, 2010; Kranstedt et al., 2006). This may depend on the communicative context (Beatty & Aboudan, 1994) or the precise nature of the co-expressive relationship between speech and gestures under investigation (Bergmann, Aksu & Kopp, 2011). Others have pointed out that stroke timing is also related to word or sentence level prosodic structures (Esteve-Gibert & Prieto, 2013; Loehr, 2007; McClave, 1994).

### Speech–gesture coordination in reception

Although several empirical studies have examined how the coordination of natural speech and gestures is processed in reception, the role and nature of this coordination for reception remains elusive. Tasks and the precise aspects of asynchrony under study play a role for results. For example, Kirchhof (2014) explicitly asked participants to judge whether video and audio tracks with different temporal shifts were synchronous or not. The stimuli included videos of natural speech and gestures but faces had been masked to eliminate compromised lip sync. The results revealed a great tolerance for temporal shifts in both directions with shifts of 600 ms being tolerated in 60% of the trials. In a similar judgement task (Leonard & Cummins, 2011), participants instead had to identify which of two versions of short video excerpts showed a single gesture that had been temporally shifted relative to its original position and to speech. The results showed that delayed beat gestures were easy to spot (even with delays as brief as 200 ms) compared to advanced gestures (unless by 600 ms or more).

Studies using electrophysiological measures of brain activity and event-related potentials (ERPs), that is more implicit measures of processing, have shown that semantic integration is affected by advancing gestures by more than 200 ms (Habets et al., 2011; Obermeier & Gunter, 2014).

These findings suggest that explicit and implicit tasks and measures may reveal different levels of sensitivity. A methodological challenge in this domain is to control speech and manipulate gesture in experimental settings, in order to truly probe effects of asynchrony on information processing.

Some studies record actors performing scripted gestures (e.g., Cassell, McNeill, & McCullough, 1999; Woodall & Burgoon, 1981). Others use video editing, combining different image sequences with the same audio track (e.g., Habets et al., 2011; Leonard & Cummins, 2011; Obermeier & Gunter, 2014), typically examining one gesture in isolation. This approach often requires the speaker’s face to be masked to avoid distraction from asynchronous speech and lip movement. Although such methods provide experimental control, they also raise methodological concerns. First, there is a risk that scripted gestures or manipulated videos draw undue attention to the objects of study. Scripted and performed gestures may have different temporal and spatial properties from naturally produced ones. It is very difficult for speakers to intentionally shift the timing of their gestures. Similarly, in videos where speakers’ faces are concealed, gestures may draw undue attention because listeners tend to predominantly gaze at the speaker’s face in normal (face-to-face) settings (Gullberg & Holmqvist, 2006). Blocked access to the speaker's face may therefore only leave listeners with gestures to look at, drawing un-due attention to them.

Another concern is that stimuli often isolate individual word–gesture combinations. However, in face-to-face communication, gestures rarely occur in isolation. Instead, they appear in sequences of sustained spoken and gestured discourse, just as words rarely occur on their own, but are surrounded by other words. Note that in the aforementioned study of explicit detection (Kirchhof, 2014), whole gesture sequences were shifted, rather that specific word-–gesture pairs. That said, although the temporal shift of an entire gesture sequence avoids the validity concerns related to isolated target gestures, it also makes it more difficult to control exactly what spoken content the shifted gesture strokes end up overlapping with. Tolerance for asynchrony might depend on addressees’ ability to relate strokes to some spoken content in the same utterance. Therefore, shifted strokes that end up with entirely unrelated speech or with silence may affect perception. To summarize, methods used to study speech–gesture coordination in reception often display a tension between experimental control and ecological validity.

As a way to address this tension, we propose an approach using 3D-animated characters based on MOCAP data of natural speech and gestures. 3D-animated characters allow us to manipulate a multitude of parameters with precision and control, and thus to study integration while avoiding some of the problems outlined above. We present how detailed control over temporal shifts of gestural strokes relative to speech can be implemented within the platform below.

### Animated characters and gestures

The use of animated characters is in itself not new in gesture research. Embodied Conversational Agents (ECAs; Cassell, 2001) are animated characters whose behavior is autonomous, i.e., not scripted or remotely controlled by a human. Their behavior is often realized by speech and gesture-synthesis, and in some cases by recombining MOCAP recordings of gestures (Xu, Pelachaud, & Marsella, 2014). ECAs were created to allow humans to interact with artificial intelligence (AI) software in the same way we interact with humans face-to-face. An interactive ECA’s behavioral repertoire should ideally include gestures. ECA development is not limited to practical applications but contributes to our understanding of natural gestures. Tools for gesture generation (synthesis) are driven by models with a basis in observations and theories of gestures (Cassell, Vilhjálmsson, & Bickmore, 2004). Evaluations of a model’s output can be indicative of its validity. Xu, Pelachaud, & Marsella (2014) asked participants to judge how similar ECA gestures were to natural gestures, and found a preference for gesture sequences that aligned with so-called *ideational units* in speech, following proposals for how natural gestures align (see Calbris, 2011). Kopp & Wachsmuth (2004) have described a method for synthesizing gestures with high-level specifications. Their gesture synthesis is constrained by empirically observed regularities in the kinematics of gestures and the coordination with speech, including adherence to McNeill’s Phonological Synchrony Rule and a stricter constraint that gesture strokes should not precede emphasized words. They compared the output of their synthesis to an actual recording, and found congruence between the artificial and natural gestures in their timing relative to speech**.** Treffner, Peter & Kleidon (2008) found that participants perceived words that were temporally overlapping with beat gestures produced by an animated character as more strongly emphasized. Another study tested learning outcomes in children listening to mathematical explanations from an ‘animated teaching agent’ with scripted gestures, either as ‘originally’ manually aligned by authors or delayed or advanced 500 ms relative to the ‘original’ alignment, and found that delayed gestures were detrimental to learning (Pruner, Popescu, & Cook, 2016) .

In experimental studies of speech–gesture integration, especially in the receptive domain, synthesized or scripted gestures may not be the best option. To address some of the methodological concerns outlined above, it may be preferable to rely on non-interactive animated characters based on MOCAP data which thus reflect natural speech and gesture production. For example, Wang & Neff (2013) asked participants to rate ‘the naturalness of the behavior’ of animated characters based on MOCAP recordings of speakers performing scripted utterances with gestures. They manipulated gestures such that the onset of the gestures was shifted relative to their lexical affiliate, varied on a discrete scale ranging from – 0.6 s (gesture before word) to + 0.6 s (gesture after word). Gestures starting after their lexical affiliates were rated as less natural when presented in parallel with gestures starting slightly before the lexical affiliate (typical in natural production). They found no difference in ratings when asynchronous videos were presented and rated in isolation. The study had an explicit focus on gestures, both in the task (comparing two videos varying only in speech–gesture timing) and in the stimuli (single, scripted gestures performed by characters with concealed faces). It therefore still remains unclear whether addressees perceive temporally shifted gestures relative to speech as unnatural when not asked explicitly about this. Because many existing methods potentially draw un-due attention to gestures, although they are rarely in focus during real-life listening, it is difficult to reliably generalize individual findings.

### Our approach

We propose and evaluate a new approach to create an experimental platform to study speech–gesture integration, while also attempting to address the tension between experimental control and ecological validity. We make use of characters that are animated on the basis of optical MOCAP recordings of natural (unscripted) human speech and gesture. The resulting digital animation data with high temporal and spatial fidelity enables us to precisely manipulate single target gestures situated in longer natural sequences of speech and gestures (ensuring ecological validity) while keeping everything else constant across conditions (ensuring experimental control).

We will present a workflow for creating animated characters based on MOCAP data, and describe in detail how we used the workflow to create experimental stimuli for an experiment designed to probe implicit detection of speech–gesture asynchrony. We will also outline how the workflow can be applied to address other research questions regarding speech–gesture reception and integration. We will discuss the results of the experiment both in terms of evaluating the method, and in terms of how they relate to previous research on speech–gesture synchrony. Finally, we will discuss potential extensions and generalizability of the method for future studies.

## Method

### Outline of workflow for a new approach

The core idea of the workflow described here is to use 3D MOCAP data from segments of spontaneous speech and gesture to animate characters, and then to manipulate those animations according to experimental variables related to the kinematics of the gesture and its relationship with speech.

**Fig. 1:**
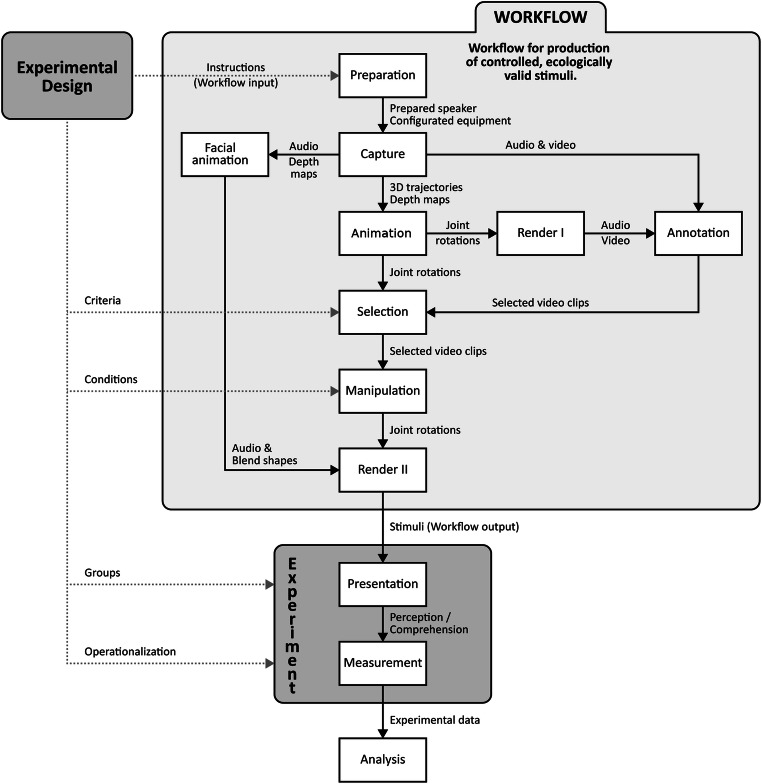
Workflow to create experimental stimuli with on animated characters based on MOCAP recordings. *Rectangular boxes* represent the steps involved. *Arrows* from the box labeled *Experimental Design* indicate ways in which the workflow can be customized to support any experimental designs testing gesture processing

Figure 1 outlines the workflow with the individual steps described in more detail below. The workflow is intended to be generally applicable to experimental designs addressing speech and gesture integration. However, we exemplify it here by an implementation of stimuli where temporal alignment is systematically varied by shifting the timing of one specific gesture within a segment of speech, while keeping speech, facial animation, and temporally adjacent movement constant.

#### Workflow: Preparation step

We used the following recording equipment: a passive marker-based optical motion capture system with eight ProReflex MCU infrared (IR) cameras, a ZOOM H4 Handy Recorder for audio recording, and an ASUS Xtion PRO LIVE 3D sensor for recording depth maps and video of the face (ASUS; www.asus.com). An additional GoPro Hero video camera 4 (GoPro; gopro.com) recorded reference videos for later use in the processing of recorded data, generation of animations, and gesture annotation. The IR cameras have a focal length of 6 mm, and can be repositioned freely.

**Fig. 2:**
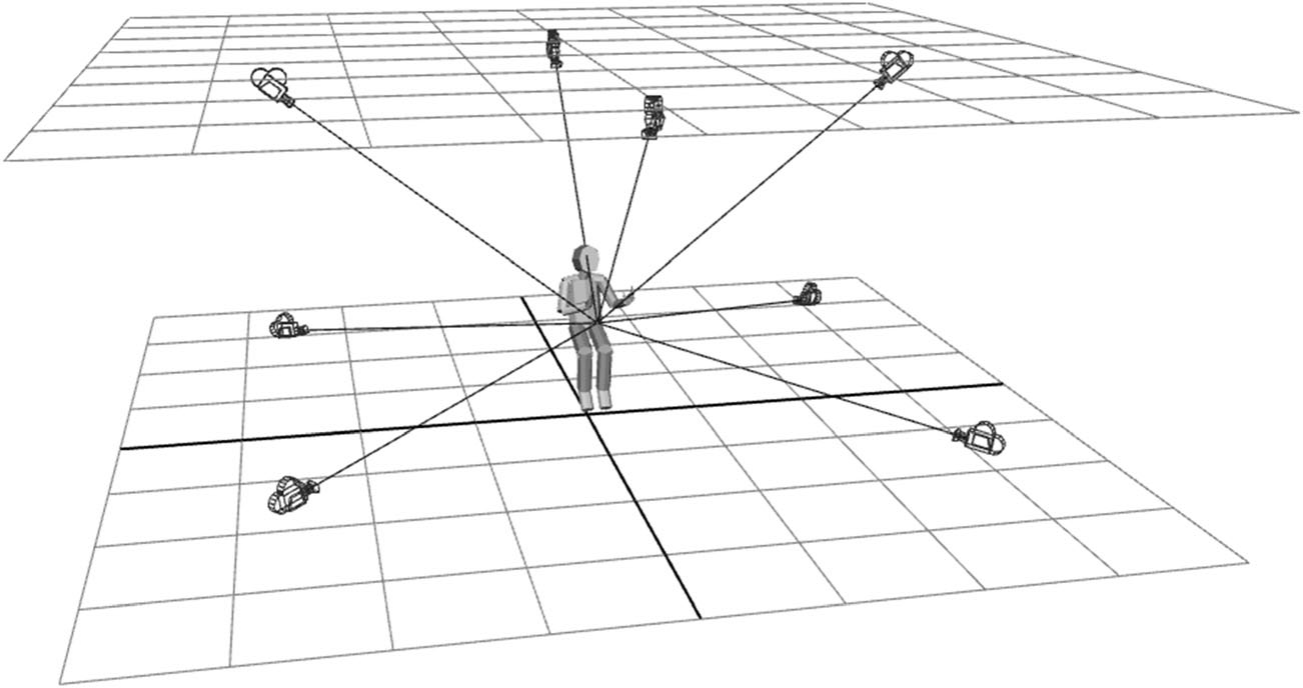
Approximate positions of IR cameras relative speakers during MOCAP recordings

Before initiating the MOCAP recordings, we conducted pilot tests to determine recording quality from different equipment setups, best spatial configuration, and equipment needed for improving data fidelity. These tests led us to some specific configurations of the MOCAP setup used for stimulus production. Full-body motion is commonly captured by cameras that are evenly distributed around the upper edges of the space, all viewing the subject from above. To reduce the risk of occluded MOCAP markers (particularly an issue for markers on the hands and the fingers), we used a customized configuration of the cameras. Taking advantage of the constraints imposed by the seated position of speakers during recordings, some cameras were placed in low lateral positions to better capture hand movements (see Fig. 2). For capturing gestures, other specific communicative situations (like a verbal presentation in front of a projected slide show) simulating the predicted range of movements in a 3D environment to find optimal camera configurations might be an option (Nirme & Garde, 2017).

**Fig. 3:**
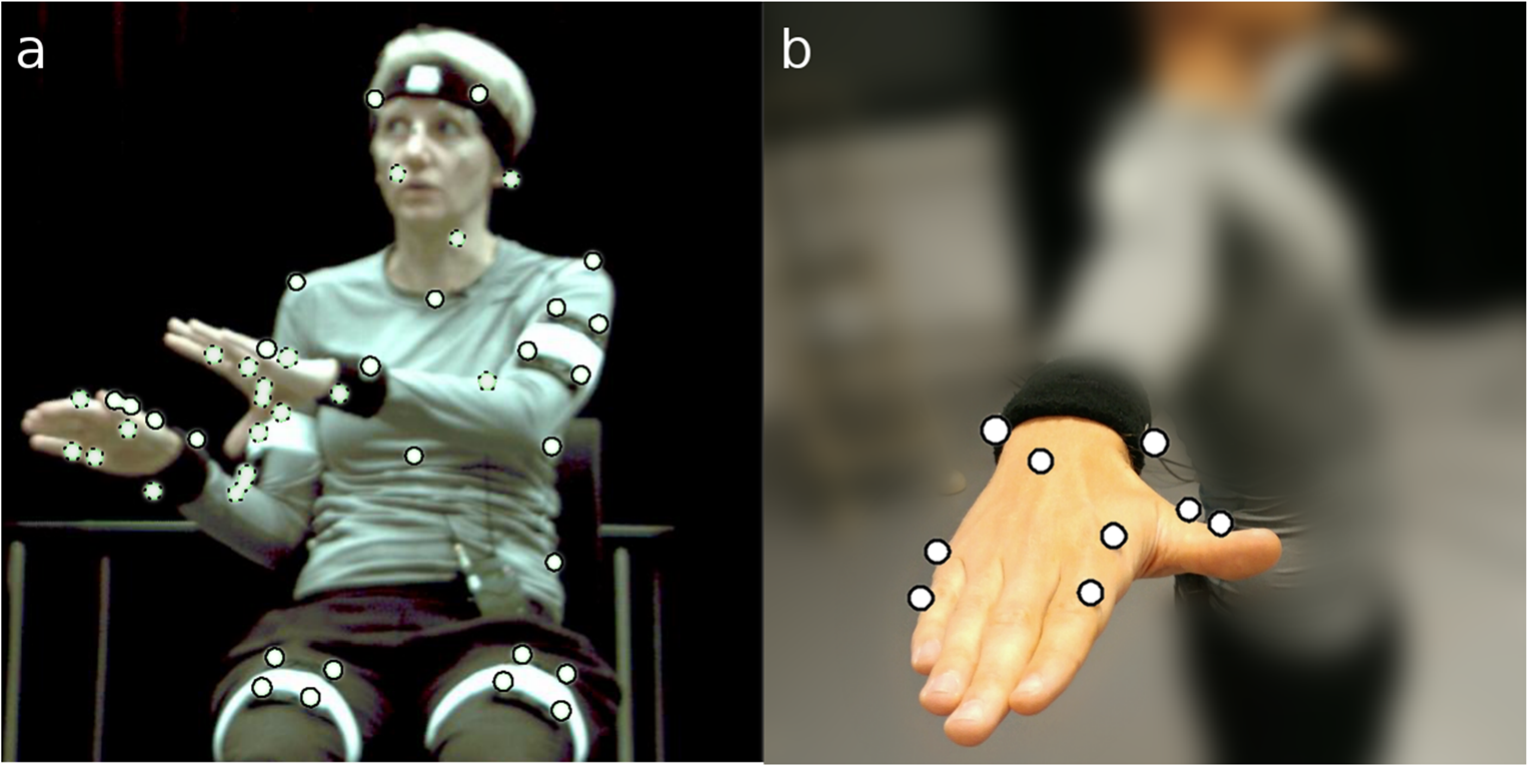
**a** Speaker during recording with optical markers at the outlines (*white circles*). *Dotted outlines* indicate markers concealed from the camera in the current view. **b** Configuration of optical markers on hands and fingers

We recruited three speakers who were told that we wanted to record them while speaking and that the recordings would be used to create 3D animated characters. No mention was made of gestures. IR reflective passive markers were attached to the head, torso, legs, arms, hands and feet of the speakers by double adhesive tape, directly to their skin, tightly fitted clothes, or elastic sweatband around the head and wrists (Fig. 3, left panel). The reflective markers were placed according to a scheme adapted from the KIT Whole-Body Human Motion Database (Mandery et al., 2015). To support reliable gesture capture, we had to modify the number of markers placed on the hands. Pilot tests had revealed that when markers are placed too close together (on all finger segments) differentiation of individual markers often failed in the post-processing, requiring time-consuming data repair. Therefore, we defined a simplified model of the hand, placing markers only on the knuckle and end of the proximal phalanx of the index and little fingers as well as the metacarpal bone and proximal phalanx of the thumb (Fig. 3, right panel). The movements of the middle and ring fingers were inferred from the adjacent index and little fingers (see *Workflow: Animation step* below). Reducing the number of markers limits the risk of unreliable differentiations of markers and estimated 3D- positions. Our custom marker set for the hands resembles Hassemer’s (2016) ‘Minimal Marker Set’, designed specifically to distinguish gestures depicting (one dimensional) ‘measures’ and ‘shapes’. Hassemer placed markers on the distal (rather than proximal) joints of the index and little finger, and at the ring finger knuckle, which gives a better approximation of the hand as a rigid body compared to the little finger knuckle. However, the difference in our marker set is motivated by the intention to capture different ranges of target movements. The markers in our set were chosen to capture a broader range of movements than Hassemer, and the set-up was deemed less likely to be occluded when speakers closed their fingers or oriented their hands palm-up. Photos of the speakers fitted with markers were made from different angles to be used as references in subsequent steps (see *Workflow: Character modeling* and *Workflow: Animation step* below).

For the MOCAP recordings, speakers were initially engaged in casual conversation in order to accustom them to the situation and make them comfortable, as well as to reduce their focus on the markers. This warm-up session also enabled us to perform equipment checks and minor adjustments. For each recording speakers were instructed to give route descriptions, describe objects (including infant toys), or retell narratives based on cartoons (including *Canary Row* frequently used in gesture studies and described in McNeill & Levy, 1982) or live action movie clips presented on a laptop. These are all standard tasks used in gesture research that provide a rich set of MOCAP recordings with a variety of spontaneous gestures. The order of the tasks was the same for all three speakers, but one of them did not complete the whole sequence due to time constraints.

#### Workflow: Capture step

Speakers were seated on a chair placed in the center of the range of the IR cameras, about 150 cm in front of a small table on which the Xtion 3D sensor and the H4 audio recorder were placed. Speakers faced a silent confederate addressee seated about 3 m in front of them. Speakers were at no point explicitly instructed (or implicitly prompted) to gesture. They generally gestured sparsely during the first recordings, but gradually increased their gesture production over time.

MOCAP 3D marker trajectories, audio, depth maps of the face, and referential video were recorded in parallel. IR-cameras and reference video recorded at 100 Hz, audio was recorded with 16-bit stereo at 44.1-kHz sample frequency, and depth maps were recorded at 30 Hz. To ensure synchronous lip facial animation, we used a clapperboard visible to all cameras with tracked markers attached at hinged clapsticks at the beginning of each recording. This enabled temporal alignment of recorded audio, MOCAP data, and reference video (with a margin of error of 10 ms) during subsequent processing and rendering. The Qualisys Track Manager software (version 2.10, 2015) was used to extract 3D movement data (series of marker positions) from the 2D frames captured by the IR cameras. The process relied on the software’s internal algorithms, but also required some manual adjustment of threshold values, labeling of markers, and reconstruction of missing or corrupt data.

#### Character modeling

We created two 3D characters with skeletal rigs to drive the animation, and facial blend shapes (specific individual face models mapped to basic facial expressions, such as mouth forms for basic phonemes, smile, closed right or left eye, raised right or left eyebrow, etc.) in Autodesk Character Generator (www.autodesk.com/products/character-generator). The anatomy (proportions) of the 3D characters was then adjusted in Autodesk Maya (www.autodesk.com/products/maya, version 2014, 2013) to match reference photos of the speakers. The last step is necessary to correctly map the recorded markers (from the Capture step) to the skeletal rig without anomalies generated in the transformation of animation data (see the Solving step below). The characters were created to match the approximate age and gender of the recorded speakers (one male, one female). To address research questions related to the appearance of the speaker while keeping the gestures constant across conditions, a wide range of characters can be created as long as they approximately match the dimensions of the recorded speakers.

#### Workflow: Animation step

The process of generating animations on rigged 3D characters from 3D marker data is called solving. For this we used the Autodesk MotionBuilder software (www.autodesk.com/products/motionbuilder, version 2014, 2013). MotionBuilder has a built-in *actor* model of a humanoid body with adjustable limbs. By mapping 3D markers to the anatomy of the actor model and its internal model of human kinematics, we enable the software to estimate the joint rotations causing the captured marker displacements. For each speaker, an actor was defined and its body segments were manually transformed (translation, rotation, and scale) to correctly align with the marker positions recorded while the speaker was in a *T-pose* (i.e., standing upright with both arms raised (abducted) to point straight out to the left / right). This alignment formed the basis of the calculation of joint rotations.

In a subsequent step, the MotionBuilder provides numerous possibilities to fine-tune kinetic relations and constraints of the human body. As an example, it is possible to generate animation data for the two fingers lacking attached markers (the middle finger and the ring finger) by adjusting the weights of an algorithm that interpolates from the two adjacent tracked fingers (the index and the pinky). Similarly, recorded or interpolated rotations of the proximal phalanges were partly propagated onto the more distal phalanges. The generated animation scheme was then transferred to the skeletal rig of a pre-modeled 3D character rig as keyframed (skeletal) joint rotations at 100 frames per second (henceforth, fps). After this, some orientations of arms and hands were manually fine-tuned. The resulting series of joint rotations were filtered to remove jittering with a low-pass Butterworth filter (Autodesk, 2016; Butterworth, 1930). The generated animation data were exported in the FBX file format (standardized format for 3D modeling and animation).

Facial animation was generated using the FaceShift Studio 2015 (now discontinued) software. The software used a pre-recorded training set of specific targets (facial expressions). Based on depth map data and video captured by the Xtion IR 3D sensor, a weighted combination of the target expressions is estimated and mapped onto the model as keyframes representing the weighted combination of *blendshapes* (target mesh deformations representing idealized expressions; Lewis et al., 2014) at a frame rate of 30 fps. The facial animation data were exported in the FBX file format. The workflow, particularly the capture step, can be simplified by generating facial animation from the recorded speech signal by inferring *visemes* (lip movements and other facial expressions associated with specific phonemes; Fischer, 1968) from phonemes detected in the speech signal (e.g., Beskow, 2003; Cohen & Massaro, 1993; Edwards et al., 2016; Pelachaud, Badler & Steedman, 1996). However, it is worth noting that there is no one-to-one correspondence between phonemes and visemes. Moreover, readily available tools mostly support English rather than other languages, including the built-in tools available in MotionBuilder.

Unless research calls for the faithful reproduction of facial expressions beyond visemes, for example affective facial expressions, it is preferable to generate facial expressions from the speech signal because it does not restrict the direction the speaker can be facing. Not having to consider the placement of a sensor or camera to capture a speaker’s face facilitates setups where speakers direct their speech to more than one addressee or shift their direction between some artifact in the environment and the addressee. If capturing facial expressions beyond visemes is a priority, there are alternatives to FaceShift that use camera and / or IR sensor input and output animation based on blendshapes or other facial rigs. Examples include Faceware (www.facewaretech.com) and f-clone (f-clone.com).

Another option is to add facial markers to the marker set described in *Workflow: Preparation step.* MotionBuilder has a built-in tool that can extract blendshape based animation, similarly to how body movement is ‘solved’ from marker data via the actor model. Deng et al. (2006) describe a method for generating mappings of marker-based facial MOCAP data to blendshape animation, based on manually training a generative model on a few facial expressions. Dutreve, Meyer & Bouakaz (2008) propose a method for transferring facial expressions based on ‘feature points’ defined in two or three dimensions on the faces of a recorded person and a character model. Three-dimensional marker-based motion capture has the advantage of not restricting the speaker to face any specific camera, but the added work in processing the MOCAP data can be time consuming, depending on the fidelity of expression needed.

#### Workflow: Render step 1

*Rendering* is the process of generating an image (or sequence of images) from 3D models, applying texture and shading effects. The basic steps preparing for rendering generally include defining texture parameters, arranging and setting light sources, and configuring a virtual camera *viewport*. In Workflow Render Step 1, the animation data from the Solving step were transferred to one of the two 3D characters using Autodesk Maya (version 2014). The facial animation data was added in the second rendering step (see Workflow: Render Step 2).

In preparation of rendering, the animated 3D characters were placed in a so-called *scene* set up with light sources and viewport properties. First, a spotlight directed at the character’s torso was placed in the scene. The spotlight was positioned with a linear drop off from the center, highlighting the character’s face and gestures while casting a soft shadow on the hands when resting in the speaker’s lap. This was a simple way to mask small anomalies in the spatial alignment between the hands and legs. Secondly, a camera was added to the scene, placed roughly in the position of the addressee relative to the speaker, with an angle of view set to 20 degrees. The characters’ main gaze directions were fixed at the center of the rendering viewport. This gaze direction does not correspond to the recorded speakers’ actual main gaze directions, but because the speakers are always moving their heads while speaking, the artificial gaze behavior is not too noticeable.

The rendering generated 1024 x 768 (RGB) images representing the camera’s view of the 3D characters against a black background using the built in Maya Hardware Renderer 2.0. Videos in this first rendering process (Render step 1) – before selection and manipulation of experimental stimuli – were rendered at 100 fps, and composed in Avidemux (version 2.5.2) for export into the gesture and speech annotation software (see Workflow: Annotation step below).

#### Workflow: Annotation step

Next, the recorded reference videos, audio recordings, and rendered videos of the animated characters (without facial animation) were imported into ELAN, an open-source multimodal annotation software (Wittenburg, Brugman, Russel, Klassmann, & Sloetjes, 2006). In preparation for the experiment, we annotated onsets and durations of dynamic gesture strokes in the reference video recordings (see section *Workflow: Preparation step*). The audio recordings were annotated independently (without video) for the onset of stressed syllables in selected experimental target words. The annotations were exported from ELAN as comma separated text files (CSV). Other experimental applications of the workflow would require different or additional annotations such as word class or gesture viewpoint, for example (McNeill, 1992; Parril, 2010). Note, however, that features related to gesture kinematics, such as handshapes (Hassemer, 2016), velocity (Trujillo et al. 2018), or similarity to gesture prototypes (Müller, Baak, & Seidel, 2009; Schueller et al., 2017) can be derived from MOCAP data using objective criteria rather than subjective annotations.

#### Workflow: Selection step

Audio recordings, reference videos, rendered animations, and annotation data were examined for the selection of suitable segments to test implicit detection of asynchrony between speech and gestures.

To be selected for the experiment, a segment had to meet the following criteria:

1. The MOCAP-based rendered animations should be of sufficient quality, and the gesture movements should be consistent with what was visible in the reference video. Approximately 10% of the captured frames of the female speaker, and 5% of the male speaker were discarded due to the face or one or more fingers being occluded.
2. It should include a *target stroke* that temporally overlapped with a stressed syllable in a *target word.* More specifically, the onset of a stressed syllable should occur within the time interval *starting at the stroke onset and lasting for the duration of the stroke.* We applied no further criteria (e.g., word class, semantic content, etc.) to the selection of target words, beyond overlap with a gesture stroke.
3. The target stroke should be surrounded by at least one other gesture.
4. There should be some temporal separation between the target gesture and preceding or following gestures.

In total, 16 segments from recordings of two of the speakers were selected (seven with a female speaker), all of which included a gesture (unrelated to the target word) preceding the target gestures, and half of which additionally included a subsequent gesture. The mean duration of the 16 videos was 9.51 s (SD 1.8 s).

To maintain a consistent link to naturally produced gestures, the first criterion regarding MOCAP data quality would have to be fulfilled. However, to address other research questions, criteria need to be adapted in accordance with the type of gesture and manipulation in focus. It is of course also possible to select rendered videos of entire recordings instead of short segments, if the focus is on how gestures affect comprehension at a global level.

#### Workflow: Manipulation step

To create stimuli for an experiment addressing the implicit detection of speech–gesture asynchrony (see section *Experiment*), we needed to shift the target gesture strokes independently from audio track and facial animations.

We defined three experimental conditions: (1) Original synchrony (G-SYNC) between gesture and speech (i.e., overlap between target gesture stroke and a stressed syllable of the target word); (2) target stroke onset advanced by 500 ms, resulting in the target stroke occurring before the stressed vowel in the target word (G-ADV); 3) target stroke delayed by 500 ms, resulting in the target stroke occurring after the stressed vowel in the target word (G-DELAY). The magnitude of the temporal shifts (500 ms relative to the original synchrony) was selected to be within the tolerated offsets (600 ms) observed in explicit detection studies (Kirchhof, 2014), but above the offsets (200 ms) observed to yield effects on ERPs (Obermeier & Gunter, 2014).

**Fig. 4:**
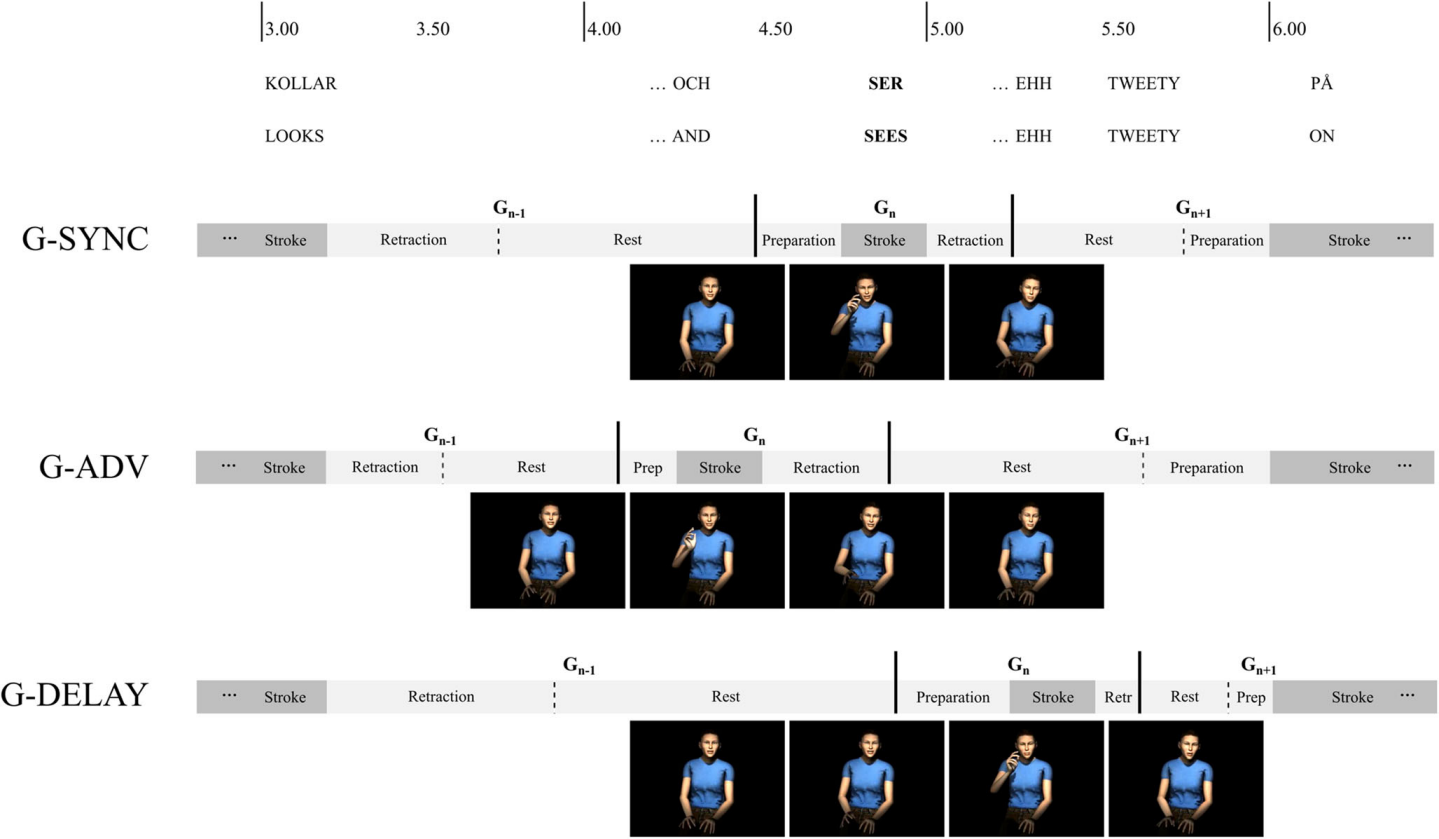
Schematic representation of an example configuration for the three experimental conditions (G-SYNC, G-ADV, G-DELAY). In the G-ADV and G-DELAY conditions, the durations of intervening gestures phases (retraction, rest, and preparation) between the target stroke and the preceding/succeeding strokes were modulated

Three versions of each of the 16 selected segments were created by prolonging or shortening the temporal duration of intervening phases between the target stroke and the preceding and succeeding strokes. The durations of target strokes, as well as onsets and durations of preceding/succeeding strokes, were kept intact (Fig. 4). To apply the manipulations systematically, we implemented a script in Autodesk Maya that read the annotation files (see Workflow: Annotation step) and shifted the target strokes.

Examples of videos including synchronized, advanced, and delayed gestures can be found in the online supplementary materials:

https://lu.box.com/s/i50n2poq2edriv4pqduoklwutp5kb6sc

https://lu.box.com/s/uu1jmbdhvrel51k2ic4ed6dnh6w5w56a

https://lu.box.com/s/ns2etws5lz5efd20r8dq6rl0jl33k47u

The 3D animation data allow for a range of other kinematic manipulations related to relevant research question about speech–gesture integration. The conceptually most basic one, to eliminate specific gestures within natural sequences, requires somewhat more elaborate kinematic manipulations, but can still fairly easily be implemented by speeding up, slowing down and blending together surrounding gestures. Manipulation of hand configurations could be implemented by replacing or blending recorded finger movements with hand configurations conforming to predefined classes. Manipulations related to gesture space or deictic features might require recalculating joint rotations by inverse kinematics (see an example of such an implementation in Ballester et al., 2015).

#### Workflow: Rendering step 2

Based on the temporal settings for the manipulation of the intermediate gesture phases in the Experimental Manipulation step, the video frames of the final experimental stimuli (animations of the 3D characters) were rendered in Autodesk Maya. After minor adjustments of the light sources and viewport settings, and addition of (non-manipulated) lip sync and facial animation data to the character, the frames (1024 x 768 images) were rendered at 25 fps. Down sampling to 25 fps was made to avoid video lag during the experiment presentation of the video material (see below), where audio-visual synchrony obviously is a central factor. After that, the audio tracks and the rendered images were mixed and encoded using the Avidemux video editor (version 2.5.2).

### Experiment (Presentation step & Measurement step)

The experiment aimed to examine whether participants detect speech–gesture asynchronies when such asynchronies appear embedded in a sequence of connected discourse (an ecologically valid setting). We used a behavioral method similar to previous studies focused on explicit perception of asynchrony (Kirchhof, 2014; Leonard & Cummins, 2011), but designed to probe implicit perception using stimuli with animated characters created in the approach outlined above. We ask the following questions:

1. Is asynchrony implicitly perceived as unnatural when gesture strokes are temporally shifted away from their original location?
2. Is implicit perception of speech–gesture asynchrony affected by the nature of the relationship between the gesture and the speech signal? That is, is it affected by whether target strokes overlap with non-target words or with pauses?

#### Participants

We recruited 32 native Swedish speakers (18 female), aged 18–57 years (*M* = 28, *SD* = 10). Each participant was rewarded a cinema ticket voucher for their participation.

#### Materials

The experimental stimuli consisted of 64 short videos of one of two animated characters (one female) speaking and gesturing. The animated characters were generated from motion capture recordings of two Swedish speakers. The 64 videos were based on 16 original selected sequences from the motion capture material, each sequence presented in four different versions, corresponding to three experimental conditions (G-SYNC, G-ADV, G-DELAY, see *Workflow: Experimental manipulation step*) and one control condition where a pitch modulation had been added to the original G-SYNC condition during a few non-target words (*Audio distorted,* A-DIST). A-DIST was included to control that participants did not attend to one modality exclusively. In addition, in the 32 videos where gestures had been temporally shifted (the conditions G-ADV and G-DELAY), we noted what the shifted gesture strokes overlapped with in speech in their new location, that is with a non-target word (25/32) or filled or unfilled pauses (7/32).

#### Design and tasks

The experimental task was a naturalness judgement task. Participants had to watch videos of 3D animated characters, and answer the question “To what degree do you think the video you just saw was based on a real speaker or generated by a computer program?” Participants responded using a mouse on visual analog scales (VAS) on the screen in front of them. The scales consistently (without counterbalancing) ranged from left to right ‘completely computer generated’ (0) to ‘completely human’ (1). The subjective judgments of the speakers in the videos as being computer or human generated was used as a metric of whether asynchrony affected participants’ perception of asynchrony, assuming that (implicit or explicit) perception leads to ‘less human’ and therefore ‘more computer generated’ (0) ratings. Participants were presented each of the 16 videos in one version (condition) only, with combinations of video and condition counterbalanced over all participants. All participants were thus exposed to all four conditions (four videos per condition), because we predicted large variance of individual participants’ distribution of VAS responses. We preferred a mixed factorial design over a pure within-subject design in order to avoid repetition of the same video, which would risk drawing explicit attention to the manipulation.

We also devised a second, explicit introspective task where participants were asked to assess how important 12 different properties (Table 1) had been for their judgements of videos as ‘computer generated’. Participants again responded using a left-right VAS ranging from ‘not at all important’ (0) to ‘extremely important’ (1). The task probed whether participants were able to explicitly pinpoint aspects of unnaturalness in the videos with regard to the manipulated target gestures, specifically speech–gesture asynchrony. All the properties and corresponding VAS (directly below the corresponding property) were presented on-screen at the same time, in two columns with six items in each. Each VAS covered approximately 25%, or 40 cm, of the screen. Only item 11 (“How important was [...] the person’s hand movements?”) was related to the manipulations and relevant for the current study. All other items were fillers and excluded from analysis.

**Table 1:** The 12 items in the introspective task

		M	SD
Item	When judging videos as ’computer generated’ I did so based on:	0.36	0.29
1	... the person’s appearance	0.59	0.31
2	... the person’s voice	0.53	0.24
3	... the person’s posture	0.31	0.33
4	... what the person said	0.45	0.26
5	... the person’s speech rate	0.58	0.28
6	... the person’s prosody	0.42	0.26
7	... the person’s lip movements	0.54	0.27
8	... the person’s facial expressions	0.48	0.27
9	... the person’s gaze	0.55	0.27
10	... the person’s head movements	0.7	0.23
11	... the person’s hand movements	0.56	0.33
12	... the person’s stiffness	0.36	0.29

Translated from Swedish. The third column includes the mean ratings of the importance of different properties for “unnatural” judgements. 0 = not at all important, 1 = extremely important

#### Procedure

Before starting, participants signed a consent form and were informed that any collected data were to be treated anonymously and that they were free to leave the experiment at any time. For the experimental task, they were instructed to indicate their ratings on a horizontal VAS following each video. If a participant asked the experimenter about what they should look at specifically, they were instructed to just go with their general impression. They first performed a practice trial, watching a video with synchronized speech and gestures.

The stimuli were projected on a 160 x 120 cm projector screen 2 m in front of the seated participants, showing the animated speaker life sized (cf. Gullberg & Holmqvist, 2006). Participants wore headphones throughout the experiment with the volume identically set for all participants. They watched videos on the screen and responded using a mouse by clicking on analog scales centered on the screen with approximately 50% horizontal extension (80 cm). After participants had rated the 16 videos in the experimental naturalness judgment task, they proceeded to complete the introspection task.

#### Analysis

All analyses were performed in R (version 1.0.136, R Core Team, 2016). We performed mixed-model linear regression analyses using the lmerTest package (Kuznetsova, Brockhoff & Christensen, 2017) and calculated coefficients of determination (marginal and conditional R2; Nakagawa & Schielzeth, 2013) using the MuMIn package (Barton, 2013).

## Results

### Experimental naturalness judgement task: implicit ratings of naturalness

The data from the experimental naturalness judgement task (*n* = 512) was analyzed using mixed model linear regression (Eq. 1), with fixed factors temporal shift of target gestures (synchronized, 500 ms before, 500 ms after) and speech content overlapping with target gestures (target word, non-target word, pause). In addition, non-experimental fixed factors were auditory distortion (0,1); presence of additional gestures (before, before and after); speaker identity (female, male); and video duration. The model included participant and video ID as random intercepts, and random slopes for temporal shift by participant and speech overlap by participant.

1 $$ \mathrm{Rating}\sim \mathrm{gesture}\ \mathrm{shift}+\mathrm{verbal}\ \mathrm{overlap}+\mathrm{auditory}\ \mathrm{distortion}+\mathrm{video}\ \mathrm{duration}+\mathrm{speaker}+\mathrm{other}\ \mathrm{gesture}\mathrm{s}+\left(1|\mathrm{participant}\right)+\left(1|\mathrm{video}\right)+\left(\mathrm{gesture}\ \mathrm{shift}|\mathrm{participant}\right)\kern0.5em +\kern0.5em \left(\mathrm{verbal}\ \mathrm{overlap}|\mathrm{participant}\right)\kern0.5em +\upvarepsilon $$

The mixed-model linear regression revealed no significant effect of temporal shift of target strokes relative to speech, whether advanced (β = .057, *t* = .838, *p* = .403) or delayed relative to target words (β = .013, *t* = 0.195, *p* = . 845). Further, there was no effect of gesture strokes overlapping with non-target words compared to overlapping with target words (β = -.026, *t* = – .382, *p* = .703). However, we did find a significant negative effect of strokes overlapping with pauses compared to strokes overlapping with target words (β = – 0.158, *t* = – 2.150, *p* = .033). That is to say, videos that contained gestures that overlapped with pauses were deemed to be less natural.

**Fig. 5:**
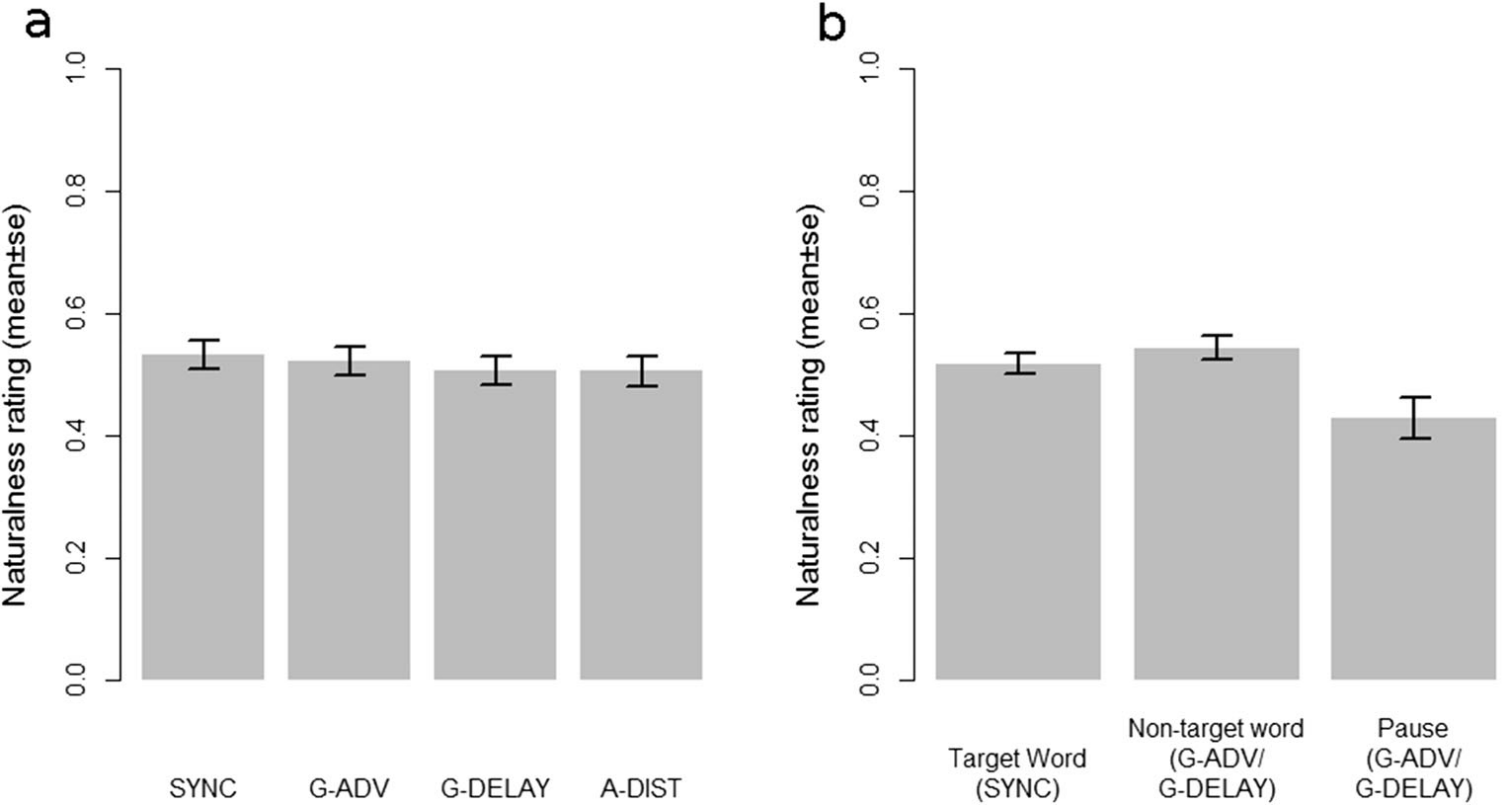
**a** Effects of experimental conditions on implicit detection of naturalness (0 = not natural; 1 = natural). G-SYNC: original speech–gesture timing, G-ADV: gesture advanced by 500 ms; G-DELAY: gesture delayed by 500 ms, A-DIST: parts of audio track distorted during non-target words. **b** Effects of speech–gesture stroke overlap with target, non-target words, or speech pauses on implicit detection of naturalness (0 = not natural; 1 = natural). G-SYNC: original speech–gesture timing; G-ADV: gesture advanced by 500 ms; G-DELAY: gesture delayed by 500 ms

Turning to non-experimental factors, there was no effect of auditory distortion in control condition A-DIST (β = – .028, *t* = 1.039, *p* = .300), of other gestures occurring before and after (as compared to only before) (β = .049, *t* = 1.071, *p* = .306), of speaker identity (β = – .028, *t* = – 0.607, *p* = .555), or of video duration (β = – .001, *t* = – .150, *p* = .883). The overall fit of the regression was estimated by marginal *R*^*2*^ = .0359 (only including fixed factors) and conditional *R*^*2*^ = .364 (including fixed and random factors). Figure 5 illustrates the results with regards to experimental conditions and speech–gesture stroke overlap. These results indicate that shifting target strokes 500 ms in either direction has no impact on how natural (humanlike) the animations are perceived to be, as long as the target strokes overlap with some spoken content.

**Fig. 6:**
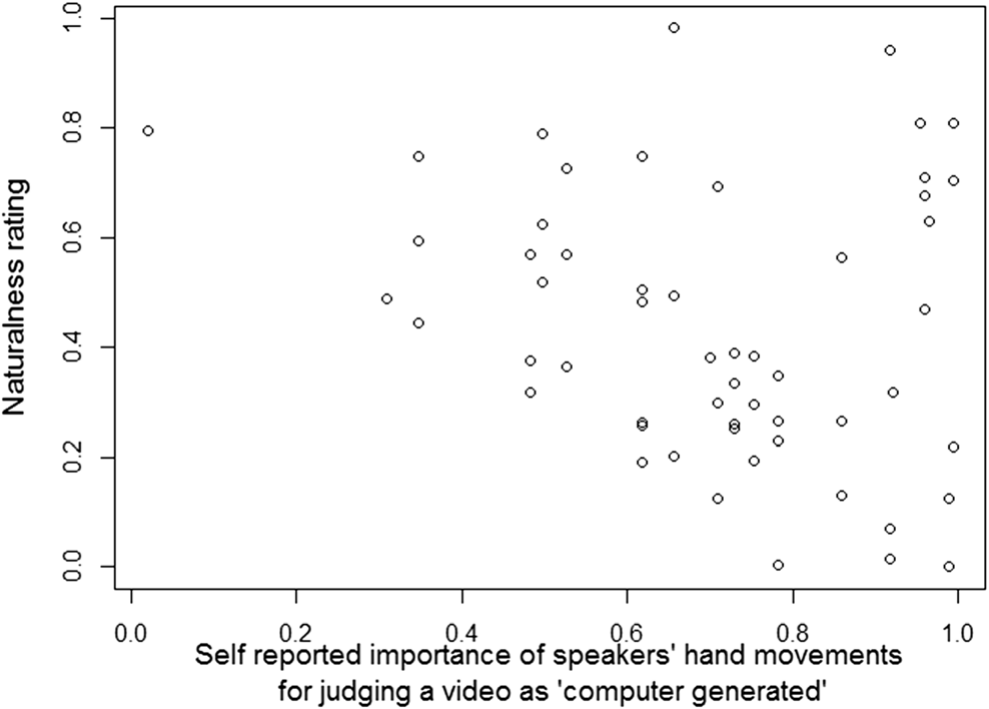
Ratings of videos where shifted strokes overlap with pauses plotted against the subsequent ratings of the importance of “hand movements” for identification of “computer generated” videos

### Introspection task

Table 1 summarizes the subjective ratings of importance of different properties of the speaker. To examine whether the implicit ratings of naturalness were related to the explicit ratings of the importance of hand movements, we performed a Kendall's rank correlation between the responses to item 11 (the importance of hand movements) and the implicit ratings of naturalness. Because the analysis of the experimental task only revealed a significant effect of shifting target strokes to overlap with pauses (and no general effect of temporal shifts), we included only the implicit ratings of naturalness to the videos with a stroke overlapping a pause (in total 56 observations). We found a significant negative correlation (Kendall's τ – .217, *p* = .021, *z* = – 2.316). In other words, the more important a participant rated hand movements to be as a basis for their judgements, the more likely they were to have judged gestures during pauses as unnatural.

## Discussion

We have outlined an approach using motion capture recordings of real speakers and their gestures to animate characters with a view to create a viable experimental platform for testing multimodal information processing. We have described a workflow for creating such animated characters to create controlled stimuli suitable to experimentally examine effects of asynchrony on speech–gesture processing. We have presented first, how we configured the MOCAP setup and instructed speakers; second, how MOCAP recordings were used to create animated characters; third, how these were manipulated to implement the experimental conditions and finally rendered.

We have also presented an experiment testing detection of speech–gesture asynchrony implicitly. We asked partcipants to rate how natural they perceived videos, some in which the natural timing of one gesture had been either advanced or delayed 500 ms, finding no significant effect of asynchrony except in the cases where the shifted gesture strokes ended up overlapping with a pause in speech. According to the Phonological Synchrony Rule (McNeill, 1992), gestural strokes that are delayed relative to their lexical or conceptual affiliate are rare in speech. Our findings are partly in line with previous results that have measured the threshold for explicit detection of asynchrony (Kirchhof, 2014). Although Wang & Neff (2013) found that gestures delayed by as little as 200 ms affected ratings of naturalness negatively, this was only the case when videos with differently manipulated gestures were presented side by side. This indicates some differences in bounda**r**y conditions for implicit and explicit perception of asynchrony. The current finding, that gesture strokes performed during pauses were perceived as less natural, is commensurate with the observation that natural gestures typical co-occur with speech and not with pauses (cf. Gullberg, 1998; Graziano & Gullberg, 2018). These results raise interesting questions concerning other recent findings suggesting that speech–gesture asynchrony may affect processing (Habets et al., 2011) and learning (Pruner, Popescu, & Cook, 2016) detrimentally.

‘Hand movements’ were rated as quite important for judging the naturalness of the videos, compared to the included fillers and correlated with judgements of gestures during pauses as unnatural. This could indicate that participants actually explicitly perceived gestures as unnatural (at least when occurring during pauses). Note, however, that we have no indication that participants were explicitly reacting to the *timing* of gestures specifically. Ratings of naturalness generally tended to the center of the VAS scale (somewhere between completely computer generated and completely human). Even the ratings of the videos presented in the G-SYNC condition averaged little over 50%, which is surprising because they were completely based on a real speaker. However, this may be caused by the unspecific formulation of the question; we avoided any explicit explanation of what *computer generated* might mean. We wanted a quantifiable measure of perceptions of something as being ‘off’ that did not require being able to specify what.

Obviously, the current results must be treated with caution because the sample is small, but they do indicate that there is more to investigate in this domain. For future studies of the implicit effects of gestures on reception, it would be advisable to control for, or at least be aware of, possible perceptual effects of strokes during pauses. At the very least, they highlight that the multitude of methods used to study these issues generate different findings, and that more studies – perhaps using the experimental platform outlined here - could be useful in resolving them.

The proposed approach has several advantages, making it possible to design implicit experimental tasks; that is, tasks where gesture kinematics can be manipulated without attention being drawn to gestures any more than in a natural listening situation. The current study serves as an example, where we could gauge participants’ perception of speech–gesture asynchrony without explicit instruction or focus on gestures and their timing; without presenting gesture–word pairs in isolation; and without concealing speakers’ faces. Instead, the stimuli were arguably ecologically valid approximations of what it is like listening to and watching a natural speaker. We recognize, of course, that animated characters are not real people. The claims of ecological validity should therefore be understood as meaning approximations of realistic sequences of speech and gestures. The experimental manipulations per se might not conform to typical patterns in natural production (e.g., delayed gestures), but to be able to precisely implement and test them in realistic contexts allows us to better pinpoint the real-world effects of following the natural patterns.

The approach is general enough to be applicable to other research questions and experimental designs. It does not rely on concealing faces, the skills of an animator, or performance of an algorithm for gesture synthesis. As illustrated in Fig. 1, the workflow is designed to enable reuse of recorded material. The stimuli described here are reusable to study other effects, such as uptake of gestural information and other manipulations on the same animation sequences are possible (see *Workflow: Manipulation step*). Like ECAs, our MOCAP-driven animated speakers allow for extensive control over how stimuli are presented. Appearances of speakers or settings, distance or angle to speakers are all variable. They can also easily be integrated in virtual reality settings, which constitute a developing research tool with great promise (Bailenson & Yee, 2005; Blascovich et al., 2002; Bohil, Alicea & Biocca 2011; Sanchez-Vives & Slater, 2005). Compared to video recordings, digitally animated speakers can be presented in three dimensions and positioned so that their gaze and gestures are directed towards the listener. Characters exhibiting realistic behavior, including gestures, recreated from MOCAP recordings can potentially increase listeners ‘social presence’ (Schuemie, 2001) in the communicative situation.

Obviously, the approach also has its limitations given the time investment of working with marker-based MOCAP. In cases where high spatial and temporal resolution of gestural form (kinematic features) is not a priority, marker-less tools such as Microsoft Kinect may be preferable (e.g., Trujillo et al., 2018). However, the multiple cameras used for marker-based MOCAP increases the range of possible movement and orientation of recorded speakers, whereas the Kinect requires speakers to face the general direction of the sensor for optimal function.

Recent development in methods based on training artificial neural networks promise to make both facial and gestural MOCAP faster and more accessible (e.g., the *Radical* commercial application: getrad.co; Bansal, Ramanan & Sheikh, 2018; Chan et al., 2018; Suwajanakorn, Seitz & Kemelmacher-Shlizerman, 2017).

For the testing of top-down models of speech–gesture production, ECAs and gesture synthesis may be more suitable (Leiva, Martín-Albo & Plamondon, 2017; Kopp, 2017; Xu, Pelachaud & Marsella, 2014). Further, the approach is not in its current form well suited to recreate and modulate gestures of avatars in real-time. The *FaceBo* framework is one example of an application of MOCAP technology for experimental research purposes that is moving more in this direction (Lugrin et al., 2016). Despite these limitations, we argue that our approach takes an important step towards allowing us to study speech–gesture integration using stimuli that are more representative of real-world multimodal language comprehension.

## Conclusions

We have presented a methodological workflow allowing us to precisely manipulate individual gestures in longer, ecologically valid, sequences of gestures based on MOCAP recordings. Using the workflow, we were able to experimentally study implicit effects of speech–gesture asynchrony in a novel paradigm, finding that addressees do not implicitly detect speech–gesture asynchrony of 500 ms in either direction unless manipulated gestures align with pauses. We have also outlined how the workflow can be adapted in other studies related to gesture processing that require both experimental control and ecological validity. The approach thus holds great promise for gesture studies, in both video and virtual reality paradigms.

### Open Practices Statement

The stimulus material created with the described method and used in the experiment is available at https://lu.box.com/s/9mvmf4b2uu92z7k4710402m2gqwc8coo. The data can be made available as a tab separated values may be made available by contacting the corresponding author. The experiment was not preregistered.

## Abbreviations

MOCAP: Motion capture
